# Understanding educational attainment disparities in Northern Ireland: the impact of pupils’ demographic characteristics and health status on post-primary outcomes.

**DOI:** 10.23889/ijpds.v9i5.1665

**Published:** 2024-09-18

**Authors:** Jill Hnatiuk, Monica Sirski, Sharmistha Mishra, Stefan Baral, Diane Gordon-Pappas, Alan Katz

**Affiliations:** 1 Manitoba Centre for Health Policy, University of Manitoba; 2 Department of Medicine, University of Toronto; 3 Department of Epidemiology, Johns Hopkins School of Public Health

Across the UK, attainment disparities remain at the forefront of education and policy debates. This is particularly evident in Northern Ireland (NI), which although is a jurisdiction of the UK, reflects a different context due to its transition to a post-conflict society and its dually selective education system (academically and religiously). In NI, post-primary attainment disparities have been reported according to pupil characteristics such as gender and socio-economic background. However, due to the lack of available data, there is limited evidence examining the influence of the health status of pupils on their educational outcomes.

This study examines the influence of a pupil’s socio-demographic profile, health status, and school-level factors on post-primary attainment, using a linked administrative dataset that combined the household Census (2011), School Census (2010-2014) and School Leavers Survey (2010-2014) for the first time. Multilevel modelling and interactions terms were conducted to consider the relative effects of physical health and mental health on attainment. In addition, the study presents a holistic approach to understanding the association between health status and education outcomes by considering self-reported health status, the presence of an illness that limits daily activities, and the presence of a physical and mental health condition.

The findings highlight that poor physical and mental health have negative associations with post-primary attainment in NI. More specifically, pupils reporting bad health, a physical or mental health condition and a limiting illness that affects their daily activities have lower educational attainment outcomes than those with no markers of ill-health (physically or mentally).

